# Supplementary figures and images for: The role of structured reporting and structured operation planning in functional endoscopic sinus surgery
Source: PLoS One. 2020 Nov 30;15(11):e0242804. doi: 10.1371/journal.pone.0242804 (PMC7703956; doi:10.1371/journal.pone.0242804)

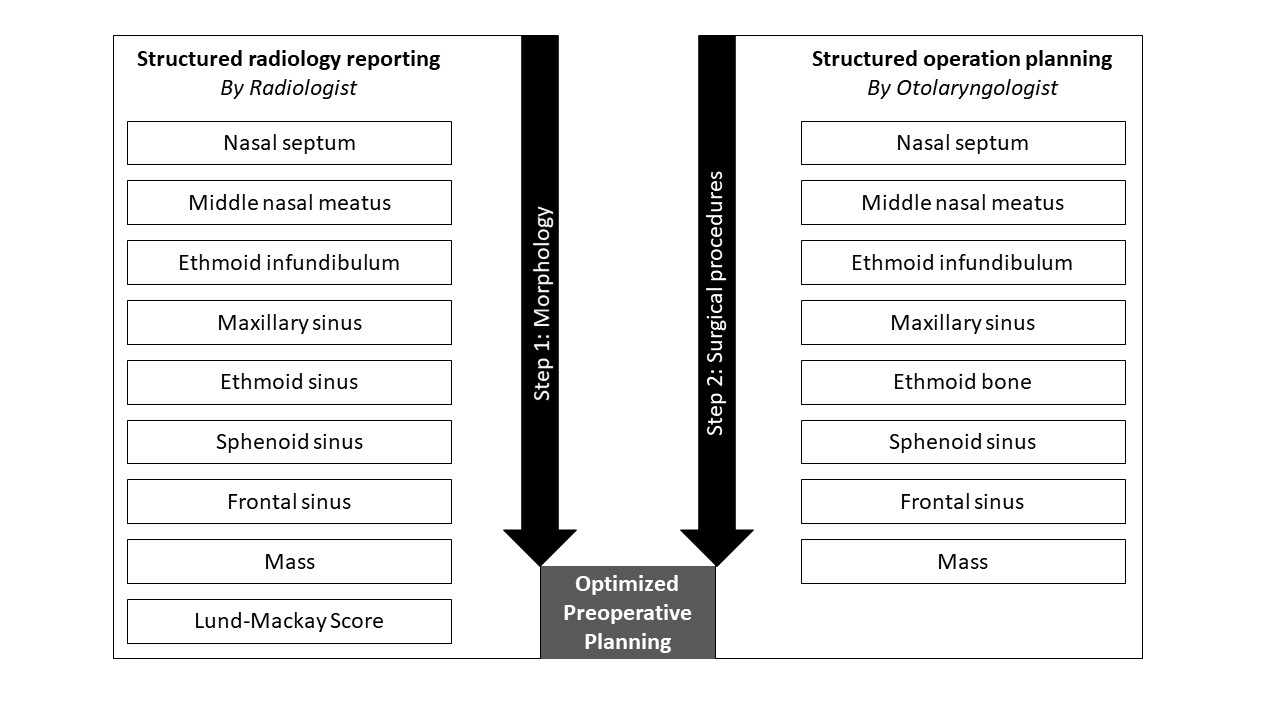

Supplement: S1 Fig — (TIF) [file pone.0242804.s001.tif]

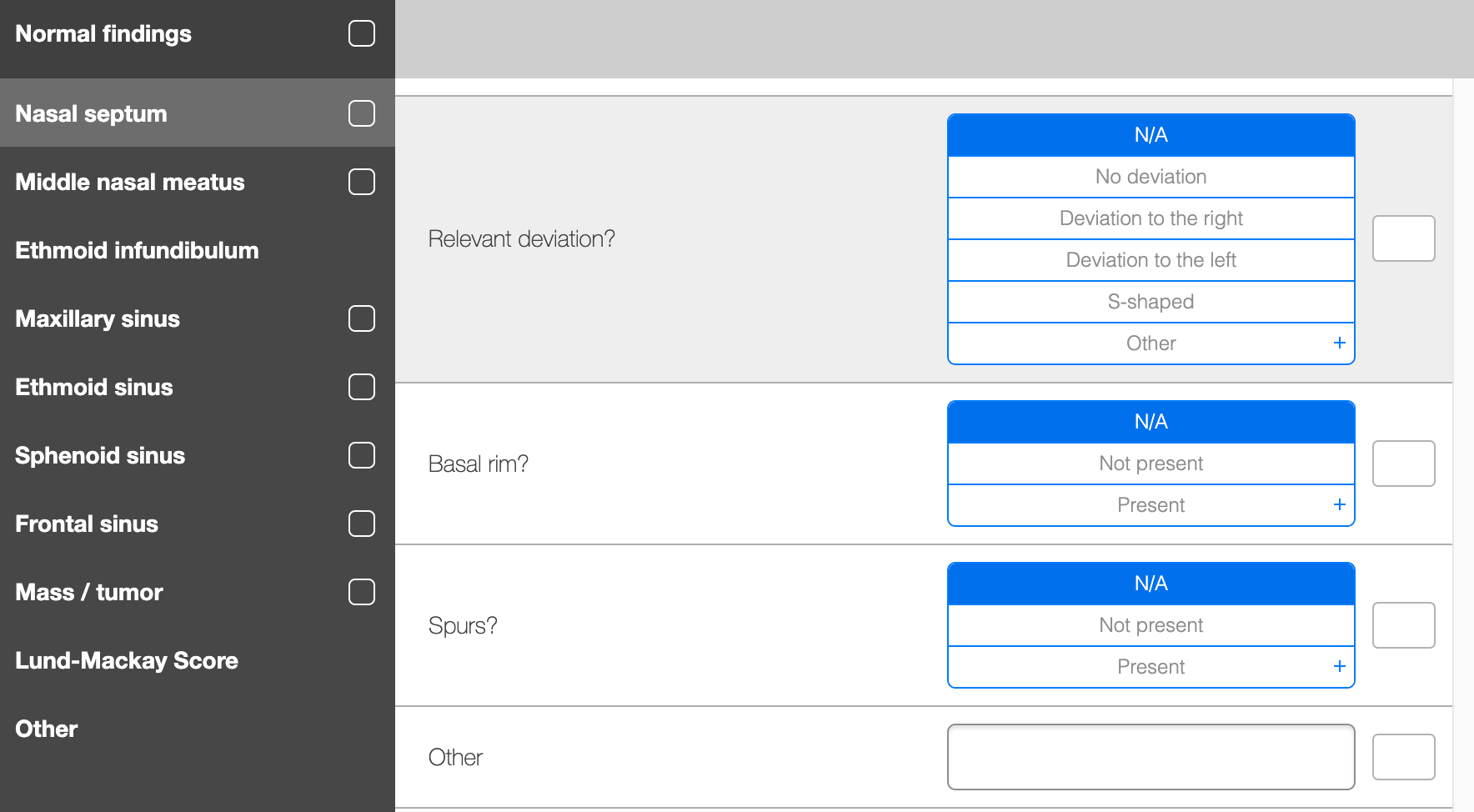

Supplement: S2 Fig — For full decision tree, refer to S1 Table. (TIF) [file pone.0242804.s002.tif]

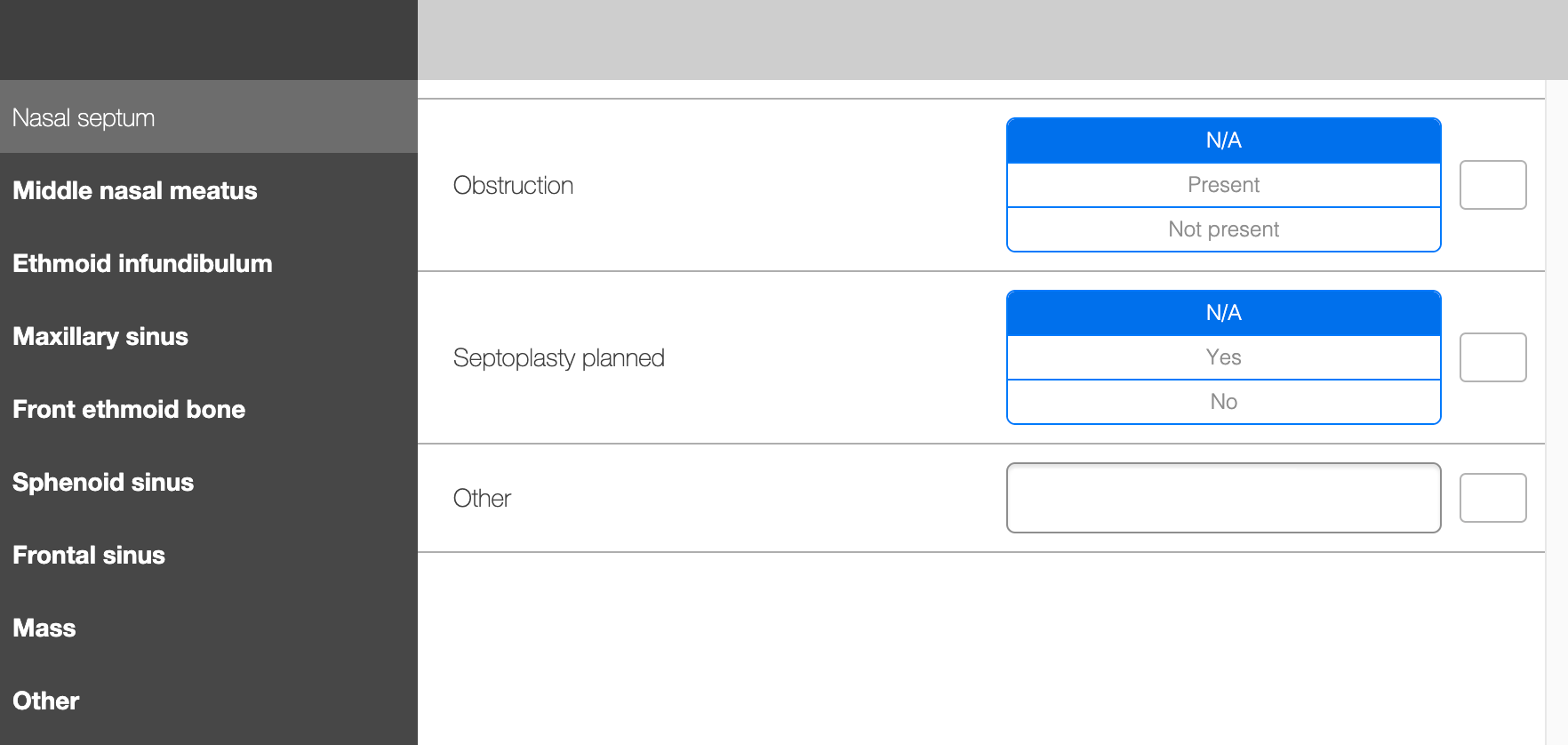

Supplement: S3 Fig — For full decision tree, refer to S1 Table. (TIF) [file pone.0242804.s003.tif]
