# Supplementary material for: The role of structured reporting and structured operation planning in functional endoscopic sinus surgery
Source: PLoS One. 2020 Nov 30;15(11):e0242804. doi: 10.1371/journal.pone.0242804 (PMC7703956; doi:10.1371/journal.pone.0242804)
Supplement: S1 Table — (DOCX) [file pone.0242804.s004.docx]

| **Nasal septum** | **Middle nasal meatus** | **Ethmoid infundibulum** | **Maxillary sinus** | **Ethmoid sinus** | **Sphenoid sinus** | **Frontal sinus** | **Mass/ Tumor** | **Lund Mackay Score** |
| --- | --- | --- | --- | --- | --- | --- | --- | --- |
| Relevant deviation? Side? | Middle nasal turbinate definable? | Projection of uncinate process? | Opacities? | Opacities? | Opacities?  Anatomy? Asymmetry? Sepation? | Development? Asymmetry? Opacities? | Presence of masses/tumors? | Automated calculation |
| Basal rim? | Concha bullosa? | Distance to orbit/ lamina papyracea? | Anatomy? Accessory ostium? | Position of middle nasal turbinate/ Ethmoid bulla? | Optic nerve? | Intra-/interfrontal cells? | Location?  Malignancy? |  |
| Spurs? | Opacities? | Opacities? | Course of infraorbital nerve? | Supre-/retrobullar recess/cells? | Internal carotid artery? | Drainage pathway of frontal sinus? | Radiologic features? Calcifications? Dental origin? Bone arrosion? Extrusion? |  |
| Free text | Free text | Free text | Position of medical maxillary wall to lamina papyracea? | Keros? Discontinuities of anterior skull base?  Anterior ethmoid artery? | Spheno-ethmoidal cells? | Anterior-posterior extension of frontal recess? | Connection to dura? Vascular tumor? |  |
|  |  |  | Free text | Discontinuities of lamina papyracea? | Free text | Free text | Free text |  |
|  |  |  |  | Free text |  |  |  |  |
