# Supplementary material for: The role of structured reporting and structured operation planning in functional endoscopic sinus surgery
Source: PLoS One. 2020 Nov 30;15(11):e0242804. doi: 10.1371/journal.pone.0242804 (PMC7703956; doi:10.1371/journal.pone.0242804)
Supplement: S2 Table — (DOCX) [file pone.0242804.s005.docx]

| **Nasal septum** | **Middle nasal meatus** | **Ethmoid infundibulum** | **Maxillary sinus** | **Ethmoid sinus** | **Sphenoid sinus** | **Frontal sinus** | **Mass/ Tumor** |
| --- | --- | --- | --- | --- | --- | --- | --- |
| Relevant deviation? Side? | Sufficient access? | Opacities? | Opacities? | Opacities? | Opacities? | Development? Asymmetry? Opacities? | Presence of masses/tumors? |
| septoplasty? | Concha bullosa? Resection? | Uncinectomy?  Conventional vs. swining door? | Maxillary antrostomy? Mucosal resection? | Anterior ± posterior Ethmoidectomy? | Optic nerve? | Frontal sinus dissection? Draf type? | Location? |
| Free text | Free text | Free text | Free text | Keros? Risk of perforation? | Internal carotid artery? | Drainage pathway of frontal sinus? | Free text |
|  |  |  |  | Dissection of anterior skull base? Discontinuities? | Free text |  |  |
|  |  |  |  | Discontinuities of lamina papyracea? |  |  |  |
|  |  |  |  | Free text |  |  |  |
